# Hair biomonitoring reveals spatial heterogeneity of multielement exposure in Bogotá schoolchildren

**DOI:** 10.1007/s10653-026-03045-7

**Published:** 2026-03-17

**Authors:** Julián David Beltrán-Ardila, Peter Alexander Escobar-Correa, Diana Angélica Varela-Martínez, Diego Armando García-García, John Alexander Benavidez-Piracón, Laura Bibiana Pinilla-Bonilla, Jefferson David Santos-Yate

**Affiliations:** 1 Faculty of Engineering, Universidad EAN, Bogotá D.C., Colombia; 2 Department of Basic Sciences, Faculty of Engineering, Universidad EAN, Bogotá D.C., Colombia; 3 National University of Colombia, Bogotá D.C., Colombia; 4 Subred Integrada de Servicios de Salud Sur ESE, Bogotá D.C., Colombia

**Keywords:** Heavy metals, Hair biomonitoring, Schoolchildren, Spatial analysis, Urban environmental health, Bogotá, Colombia

## Abstract

Children in large Latin American cities may experience concurrent exposure to multiple environmental metals. This study quantified hair concentrations of lead (Pb), manganese (Mn), cadmium (Cd), mercury (Hg), and copper (Cu) in Bogotá schoolchildren to assess spatial heterogeneity across four administrative localities.Query In a cross-sectional design, 69 pooled scalp-hair samples from 14 public schools were washed, digested, and analyzed using atomic absorption spectrometry (flame, graphite furnace, and cold vapor, as applicable). Key quality control steps included blanks, certified-reference materials, matrix-spike recoveries, and instrument triplicates; batches not meeting precision criteria were reanalyzed or flagged (see Supplementary Tables S2–S9). Missing data were handled via multiple imputations. Inter-locality differences were assessed with Kruskal–Wallis tests, and multielement patterns compared using PERMANOVA. Statistically significant differences were found for Pb, Mn, and Cd (p < 0.05), with higher levels in Bosa and, to a lesser extent, Usaquén. Hg levels were low and spatially uniform. These findings support the use of school-based hair biomonitoring to detect spatially structured metal exposure and guide targeted environmental-health actions in urban settings.

**Supplementary Information:**

The online version contains supplementary material available at 10.1007/s10653-026-03045-7.

## Introduction

Heavy metals remain a public-health concern in rapidly urbanizing settings where dense traffic, mixed residential industrial land use, and uneven enforcement of environmental standards sustain chronic exposures. Children are disproportionately affected because of age-related physiology and behavior; even low-level exposure to neurotoxicants has been linked to measurable losses in cognitive function and changes in behavior (Grandjean & Landrigan, 2006; Lanphear et al., 2005). When exposures involve multiple elements, most notably Pb, Mn, Cd, Hg, and Cu, risks may be additive or synergistic, with implications for attention, memory, motor performance, and executive function (Grandjean & Landrigan, 2006; Sanders et al., 2015). Although Cu is essential, imbalances can amplify oxidative stress, particularly in the presence of other metals (Gaetke & Chow, 2003). Hg exposure in urban populations often reflects diffuse, non-local pathways such as dietary intake, further complicating risk assessment (Grandjean et al., 2010).

From a geochemical perspective, urban soils and road dust act as carriers and reservoirs of potentially toxic elements that can be resuspended, deposited, and re-entrained into the air and onto contacted surfaces. In Bogotá and other Latin American cities, studies have documented elevated metal levels in surface dust and sediments within high-traffic districts and socio-economically vulnerable neighborhoods (Franco-Rivera et al., 2022; Peña-Fernández et al., 2014; Rodríguez et al., 2015). Linking these geoenvironmental gradients to human exposure requires biomarkers that capture recent cumulative intake and are practical for use in schools and community settings.

Hair is increasingly used as a biomarker of medium-term metal exposure in children due to its non-invasive collection and integrative nature. However, its use requires rigorous decontamination, standardization of protocols, and careful interpretation given the potential influence of dietary intake, external deposition, and hair characteristics (Esteban & Castaño, 2009; Rodríguez Martín et al., 2015; Schulz et al., 2012). The present study adopts this conservative interpretative stance while leveraging the advantages of hair biomonitoring for spatial screening in urban environments (Apostoli et al. 2006). Hair has been applied to characterize metal burdens in children and adolescents across diverse geochemical contexts, including settings influenced by both natural bedrock and anthropogenic activities, underscoring its value for environmental-health surveillance (Heng et al., 2022; Kousa et al., 2022; Varrica et al., 2022). Despite this, hair biomonitoring remains underutilized in Latin America relative to the scale of urban environmental challenges (Franco-Rivera et al., 2022; Peña-Fernández et al., 2014).

The present study applies hair biomonitoring to evaluate spatial heterogeneity in multielement exposure among schoolchildren in Bogotá. Concentrations of Pb, Mn, Cd, Hg, and Cu were measured in pooled hair samples from public schools located across administrative localities that differ in traffic intensity and socio-environmental vulnerability. Non-parametric and multivariate analyses were used to assess between-locality differences and delineate multimetal profiles. The aim is to generate locality-level evidence linking urban geochemical patterns to children’s exposure burdens and to provide a practical screening framework to inform targeted environmental-health interventions.

This contribution is novel in combining school-based pooled hair biomonitoring with spatially disaggregated multielement analysis in Bogotá, a Latin American megacity with limited prior research on environmental exposures. This study provides high-resolution locality-level data and demonstrates the feasibility of integrating biomonitoring into public school systems in Latin American megacities to support environmental-health surveillance and targeted interventions.

## Materials and methods

### Study design and participants

**Fig. 1:**
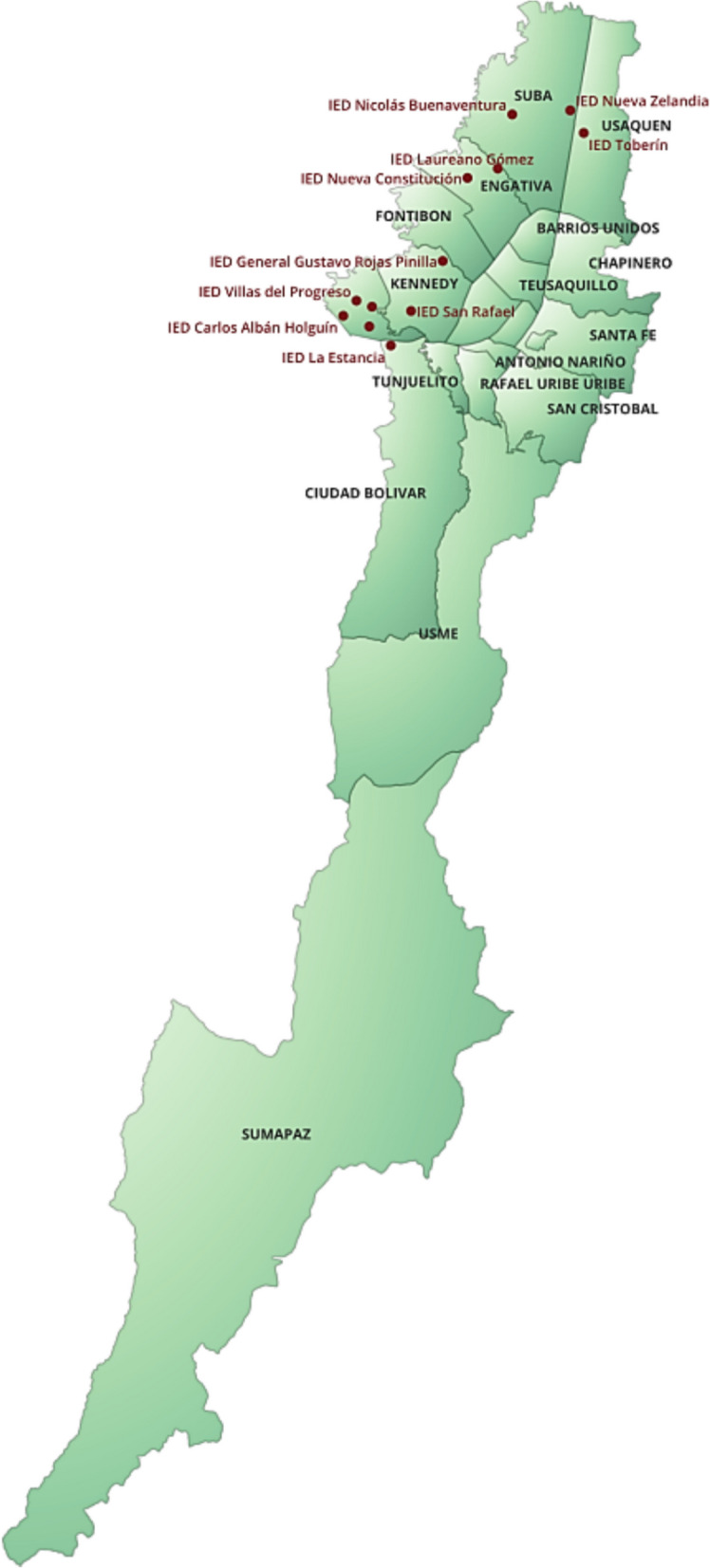
Geographic location of the 14 public schools included in the study, distributed across Bogotá’s administrative localities

This cross-sectional biomonitoring study evaluated locality-level patterns of multielement exposure among schoolchildren enrolled in public schools in Bogotá, Colombia. Fourteen schools were purposively selected across administrative localities to capture contrasting geo-environmental and traffic contexts, considering school size, accessibility, and socio-environmental vulnerability. Figure 1 presents the geographic distribution of the 14 participating schools across Bogotá’s administrative localities. These areas were selected to represent a range of environmental conditions including varying traffic density, proximity to industrial activities, and disparities in green space and infrastructure. Bosa and Ciudad Bolívar, for example, are characterized by mixed residential and industrial land use and limited vegetation cover, while Usaquén includes areas of higher socioeconomic status and greener surroundings. This stratification allowed for exploration of spatial contrasts in potential environmental metal sources.

Within each school, hair was collected under a standardized protocol and pooled at the classroom or grade level to minimize participant burden, ensure adequate analytical mass, and stabilize within-class variability. A total of 69 pooled samples were obtained. Because individual demographic data were not recorded, age and gender could not be incorporated into the statistical analyses. However, all sampled children were enrolled in grades 2 through 4, corresponding approximately to ages 7–10 years, with a presumed gender balance across classrooms given public school enrollment norms.

Eligibility criteria included enrollment at the school during the study period (February 2023–June 2024), written informed consent from parents or legal guardians, and assent from children when applicable. To limit analytical interference and external carryover, dyed or chemically treated hair was excluded, and decontamination was applied before digestion and analysis. These procedures are consistent with best practice for hair biomonitoring and with concerns about external contamination reported in the literature.

The target sample size of 14 schools and 69 classroom-level pools was planned to secure city-wide coverage of contrasting micro-environments and adequate precision for between-locality contrasts in mixture concentrations, assuming a modest design effect due to clustering. Analyses were conducted at the pool level and statistical models accounted for clustering by school or locality and right-skewness of concentration distributions (see Sect. "Data management and statistical analysis" Statistical analysis). This design aims to delineate spatial heterogeneity in environmental metal loads at the school or locality scale rather than to support individual-level inference. Our reporting follows the STROBE recommendations for cross-sectional studies. The completed checklist is provided in Supplementary Information (Table S1).

To situate the approach, recent work underscores hair as a useful matrix for medium-term exposure screening in children and urban settings, while highlighting the need for robust decontamination, quality control, and cautious interpretation with respect to diet and external sources. We adopt that conservative stance here. Each classroom-level pool was composed of scalp hair from three to six children, with a median of five per pool, randomly selected within each classroom to minimize selection bias. Equal mass, approximately 20–25 mg, was contributed per child to ensure proportional representation in the final analytical aliquot. This pooling approach was designed to optimize analytical efficiency, reduce participant burden, and stabilize within-class variability. While pooling improves logistical feasibility in large-scale biomonitoring, it precludes assessment of individual-level variation. Potential biases from differing sex or age compositions across classrooms were minimized by random sampling and consistent pooling procedures but cannot be entirely ruled out and are addressed in the study limitations.

A schematic overview of the multi-step analytical workflow, from school selection through to statistical analysis, is presented in Supplementary Figure S1C.

### Hair sampling and decontamination

Hair from the occipital region (~ 1–2 cm from the root) was collected using stainless-steel scissors cleaned between samples. Approximately 100–150 mg of hair per pool were obtained and stored in labeled polyethylene bags. To minimize external contamination, samples were washed in a 1.0% (w/v) Triton X-100 solution for 15 min in an ultrasonic bath, rinsed with Milli-Q water, immersed in 10 mL of 1.0 N HNO_3_ with 10 min of ultrasonication, and rinsed again with Milli-Q water. Cleaned samples were transferred onto cellulose filter paper (Ø 110 mm), dried at 70 °C for 4 h under dust-free conditions, cut into small fragments, and homogenized. All procedures were performed with powder-free gloves and acid-washed labware to prevent trace-metal contamination.

Sample digestion. Approximately 80 mg of dried hair (duplicate aliquots) were weighed into glassware pre-cleaned with 1.0 N HNO_3_. Mineralization was performed by adding 1.5 mL concentrated HNO₃ and heating at 105 °C for 45 min, followed by addition of 3.0 mL H_2_O_2_ (30%) and continued heating at 105 °C for 50–60 min until discoloration was complete. Digests were cooled, quantitatively transferred, and brought to 10 mL with ultrapure water (final acid ≥ 1% v/v HNO_3_) prior to AAS determinations. Procedural blanks accompanied each batch.

### Sample dissolution, instrumental analysis, and quality assurance/quality control (QA/QC)

#### Sample dissolution

After the multi-step washing protocol, ~ 80 mg aliquots of scalp hair were transferred to acid-cleaned PTFE vessels and subjected to closed-vessel acid digestion with ultrapure HNO3 (and H_2_O₂ as needed) at controlled temperature until complete clarification. Digests were brought to volume with 18.2 MΩ·cm water in acid-washed polypropylene tubes and stored at 4 °C until analysis. Procedural reagent blanks accompanied every digestion batch.

#### Instrumental analysis

Pb, Mn, Cd, Hg, and Cu were quantified using a Shimadzu AA-7000 atomic absorption spectrometer operated with element-specific lamps and optimized parameters. Cu and Mn were measured by flame AAS; Pb and Cd by graphite-furnace AAS with matrix modifiers as per manufacturer recommendations; Hg was determined by cold-vapor AAS. Multi-point external calibration was performed for each analyte within validated linear ranges; coefficients of determination were typically ≥ 0.98–0.998 (see calibration plots and regression metrics in Fig. S1A and Table S2). Instrument stability and drift were checked with mid-level calibration verification standards every ~ 10 samples; carry-over was assessed with rinsing blanks.

#### Quality control and acceptance criteria

All samples were injected in instrumental triplicate. A priori precision required ≤ 20% relative standard deviation (RSD) at the sample level; raw triplicates and RSDs are provided in Table S3. Method detection limits (LOD) and limits of quantification (LOQ) were calculated as 3.3 σ_blank/slope and 10 σ_blank/slope, respectively, using procedural blanks and the corresponding calibration slope (Tables S4 and S5). Accuracy was evaluated with a human-hair certified reference material (Hg, Cu) and matrix spikes for Pb, Cd, and Mn at two–three fortification levels per batch; CRM bias and recoveries are summarized in Table S6, and element-specific spike recoveries in Table S7. Procedural reagent blanks accompanied every batch and were required to be < LOQ; in two Pb batches, blank levels slightly exceeded LOQ. Those batches were re-run when feasible, or Pb results were blank-corrected and flagged in the dataset (Tables S5 and S8–S9). All QA/QC outcomes—calibration, sensitivity, blanks, LOD/LOQ, CRM/spikes, and triplicate precision are compiled in Figure S1A and Tables S2–S8. To assess the robustness of Pb-related findings, a sensitivity analysis was performed excluding the two digestive batches in which procedural reagent blanks slightly exceeded the LOQ. These batches corresponded to pools from Bosa (Batch ID: A12) and Kennedy (Batch ID: B03), which were either reanalyzed or blank-corrected and flagged. Excluding these batches yielded similar spatial patterns and statistical significance for Pb distributions, supporting the stability of the results. Details on the affected batches, blank levels, and recalculated values are provided in Supplementary Table S9.

### Sample digestion

A pseudo-total acid digestion was performed following a modified EPA 3050B protocol. Hair samples (approximately 0.1 g) were digested in open polypropylene tubes using 3.0 mL of HNO_3_ (70%) and 1.0 mL of H_2_O_2_ (30%) at 85 °C on a controlled hotplate for 2 h. Tubes were then cooled to room temperature and centrifuged at 3000 rpm for 10 min. The supernatant was transferred and brought to a final volume of 10.0 mL with ultrapure deionized water, yielding the digested solution.

### Sample dilution and instrumental analysis

Dilution steps were performed immediately prior to instrumental analysis, as required, to bring concentrations within the working range of each metal-specific protocol. Flame atomic absorption spectrometry (FAAS) was used for Cu and Mn, graphite furnace AAS (GFAAS) for Cd and Pb, and cold-vapor AAS for Hg. All dilutions used ultrapure water and acid-matched matrices to minimize analytical interference. Calibration included multi-point standards (R^2^ ≥ 0.98), blanks, certified reference materials, and spike recoveries.

### Data management and statistical analysis

Metal concentrations (µg/g hair) were summarized at the school and locality levels. Missing data were addressed using multiple imputations with chained equations under a missing-at-random assumption, including all metals and relevant covariates to stabilize estimates. Distributional characteristics were inspected visually. Between-locality differences were tested using the Kruskal–Wallis test with appropriate post hoc comparisons and multiplicity adjustment. Overall multimetal exposure profiles were evaluated using permutational multivariate analysis of variance (PERMANOVA) on an appropriate distance matrix. Effect sizes and pseudo-F statistics with 999 permutations were reported. Analyses were performed in Python (version 3); analysis scripts are available upon request. Pairwise associations among metals were explored using Spearman’s rank correlation on pooled-sample concentrations.

### Spatial analysis

School locations were mapped and locality-level metal burdens were summarized using a geographic information system. Spatial heterogeneity was explored with locality-level choropleths and, were informative, interpolated surfaces limited to the convex hull of sampled areas. Global spatial autocorrelation (e.g., Moran’s I) was examined for standardized metal indices. All maps include legends, scale bars, and north arrows; coordinate reference systems are reported in the figure captions.

### Ethics

This observational study involved deidentified human scalp hair. The research protocol was reviewed and approved by the Research Ethics Committee of the Subred Integrada de Servicios de Salud Sur E.S.E, Bogotá D.C., Colombia (Approval No. R-246, dated February 24, 2023). Written informed consent was obtained from parents or legal guardians, and child assent was secured where appropriate.

### Reporting guideline

This study adheres to the STROBE statement for cross-sectional studies; the completed checklist is provided in Supplementary Information (Table S1).

## Results

### Descriptive metal concentrations

A total of 69 pooled scalp-hair samples were collected from 14 public schools strategically selected across Bogotá’s administrative localities. The sampling framework aimed to capture variability in urban environmental conditions, including differences in traffic intensity, industrial proximity, and green space coverage. Each pooled sample represented 10–12 children from a single classroom, forming a locality-level exposure indicator.

**Fig. 2:**
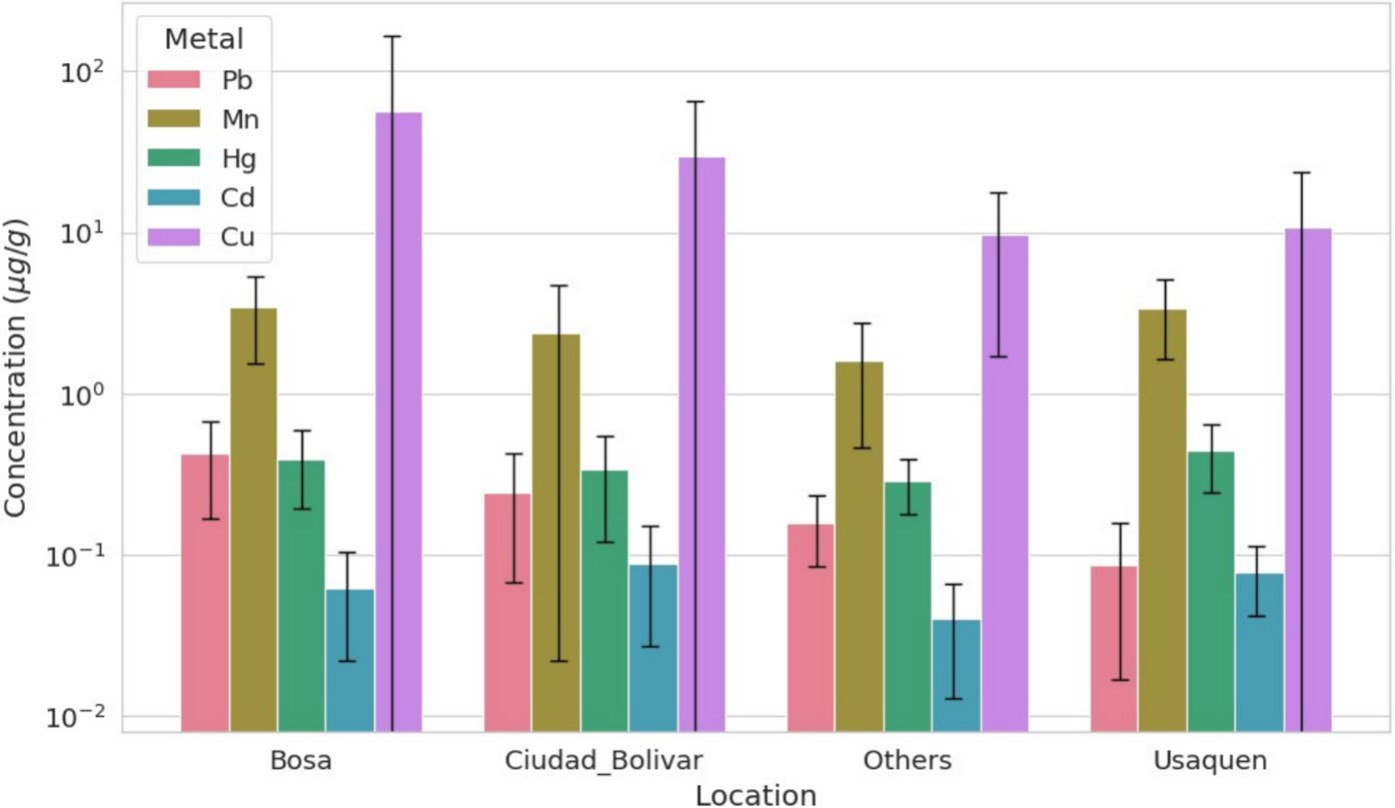
Boxplots of hair metal concentrations (Pb, Mn, Cd, Hg, Cu) by locality. Boxes show the interquartile range (IQR), the line denotes the median, whiskers extend to 1.5 × IQR, and points indicate outliers

**Fig. 3:**
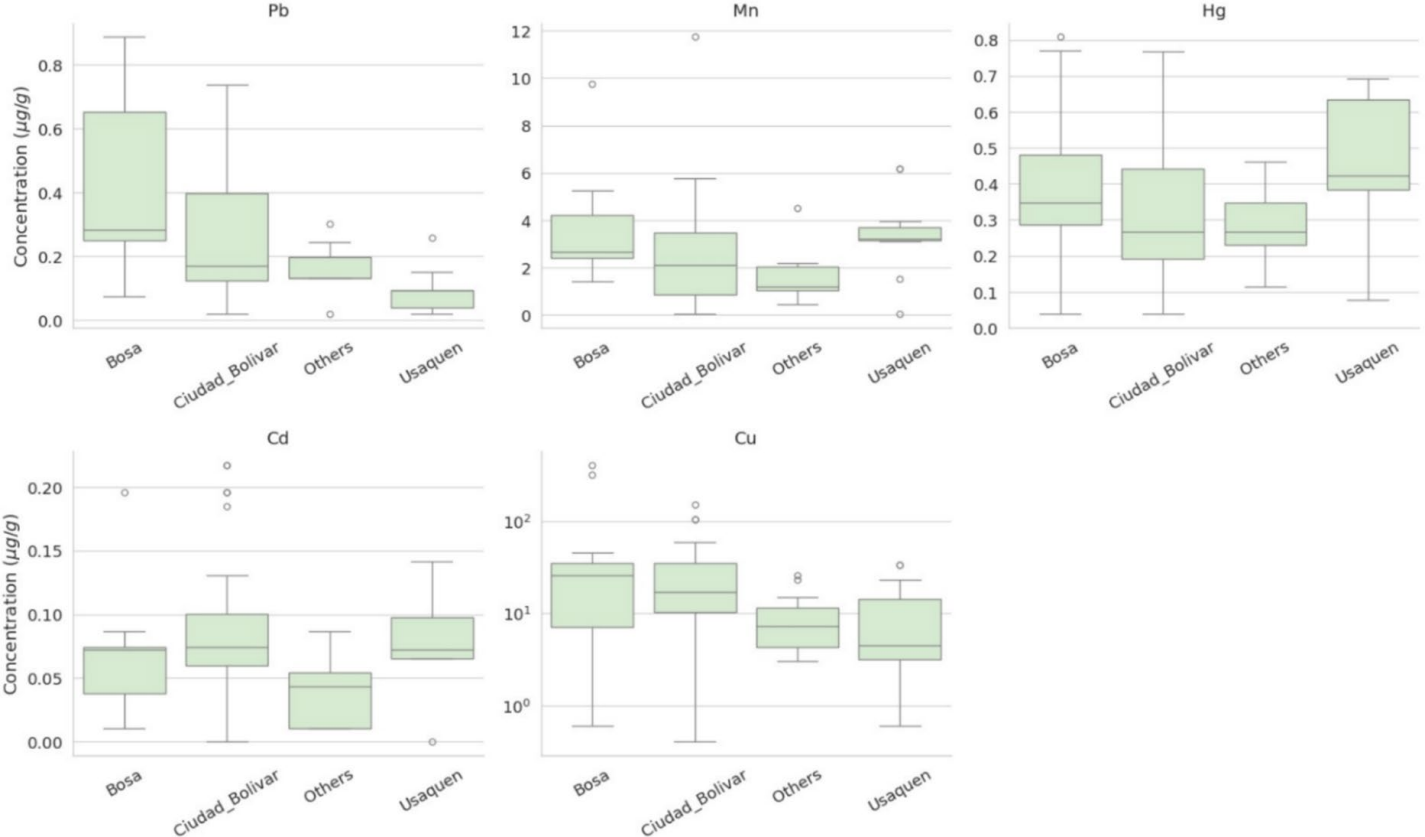
Violin plots with embedded box summaries for hair metal concentrations by locality, illustrating distributional shape, dispersion, and skewness

Descriptive statistics for the five trace metals Pb, Mn, Cd, Hg, and Cu are summarized in Figs. 2 and 3. Figure 2 displays the median and interquartile range (IQR) of concentrations (µg/g) by locality, with whiskers extending to 1.5 × IQR and individual outliers plotted. Hair metal levels showed substantial variation across localities, with notably elevated and more variable Cu concentrations in Bosa and Ciudad Bolívar. The remaining elements showed heterogeneous patterns city-wide.

Distributional patterns were further examined using violin plots (Fig. 3), which revealed positive skewness, long upper tails, and multiple outliers across most metals. These features support the use of non-parametric statistical methods for comparing concentrations across localities.

### Inter-locality statistical differences

Statistical comparisons confirmed significant differences in hair metal concentrations across administrative localities. Kruskal–Wallis tests (Table 1) indicated significant locality-level variation for Pb (H = 24.530, p < 0.001, η^2^ = 0.331), Mn (H = 12.898, *p* = 0.005, η^2^ = 0.152), Cd (H = 10.788, *p* = 0.013, η^2^ = 0.120), and Cu (H = 18.453, *p* < 0.001, η^2^ = 0.238). In contrast, differences for Hg were not statistically significant (H = 4.733, *p* = 0.192, η^2^ = 0.027). Post hoc comparisons (Dunn’s test with Bonferroni adjustment) identified significantly elevated Pb and Mn in Bosa relative to multiple localities, and moderately higher Mn in Usaquén.

**Table 1:** Kruskal–Wallis tests for between-locality differences in hair metal concentrations

Metal	H statistic	*p*-value	η^2^
Pb	24.530	< 0.001	0.331
Mn	12.898	0.005	0.152
Cd	10.788	0.013	0.120
Hg	4.733	0.192	0.027
Cu	18.453	< 0.001	0.238

**Fig. 4:**
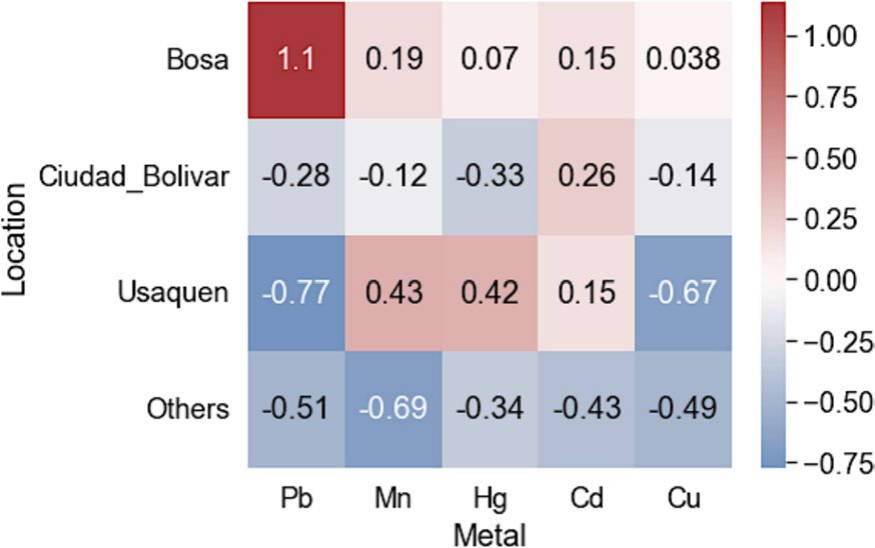
Heat map of standardized median hair concentrations (Pb, Mn, Cd, Hg, Cu) by locality; higher values indicate greater relative burden

To assess cumulative exposure patterns, a permutational multivariate analysis of variance (PERMANOVA) was performed on centered and standardized metal concentrations. Results showed significant multimetal differentiation across localities (pseudo-F = 5.1279, *p* = 0.003, 999 permutations; Table 2). The corresponding heat map (Fig. 4) highlighted locality-specific profiles: Bosa exhibited consistently elevated values for all five metals, Ciudad Bolívar was characterized by high Cd, Usaquén showed comparatively higher Mn and Hg, and the "Other" group displayed uniformly lower concentrations. These patterns delineate spatial clustering and support the presence of locality-level exposure gradients that may inform targeted follow-up efforts.

**Table 2:** PERMANOVA results for multimetal exposure profiles by locality (999 permutations)

pseudo-*F*	*p*-value	permutations
5.1279	0.003	999

## Summary of analytical performance

All analytical procedures adhered to internationally recognized QA/QC standards for trace metal determination in biological matrices. Multi-point calibration curves exhibited strong linearity (R^2^ = 0.980–0.998; Table S2), and instrument performance was verified with mid-level calibration checks. Method detection and quantification limits (LOD/LOQ) were calculated per analyte (Table S4), and procedural blanks confirmed minimal contamination (Table S5).

Instrumental precision was assessed through triplicate injections, with ≥ 90% of samples meeting the a priori criterion of ≤ 20% RSD (Table S3). Accuracy was evaluated using certified reference material (CRM) for Cu and Hg, and matrix spike recoveries for Pb, Mn, and Cd across multiple batches (Tables S6 and S7). Most elements showed recoveries within the 80–120% acceptance range. However, mercury (Hg) exhibited partial under-recovery (70–92%) in two of three spike replicates (Table S7), leading to qualified interpretation and cautionary flagging in the dataset.

Two Pb batches had procedural blanks slightly exceeding the LOQ; affected values were either reanalyzed or blank-corrected and flagged (Table S9). A sensitivity analysis confirmed that exclusion of these batches did not materially alter Pb spatial trends.

Overall, the combined QA/QC outcomes support the validity of the reported spatial patterns. Full documentation is provided in Supplementary Tables S2–S9 and Figure S1.

## Discussion

This work presents a spatially resolved framework to screen for potential hotspots of multielement environmental contamination in Bogotá, Colombia, using hair samples from schoolchildren as an integrative indicator. The objective was to characterize locality-level exposure patterns by quantifying Pb, Mn, Hg, Cd, and Cu in composite samples from strategically selected public schools and by applying non-parametric and multivariate analyses. The approach aligns with growing evidence that children are sensitive sentinels of environmental metal exposure due to age-related physiology and behavior (Bellinger, 2008; Menezes-Filho et al., 2009; Sanders et al. 2009).

From a health-relevance standpoint, median Pb levels in Bosa (1.22 µg/g) exceeded the ATSDR’s suggested reference value of 1.0 µg/g for children’s hair, suggesting a potential concern. In contrast, Hg levels remained below 0.3 µg/g across all localities, well under the German HBM-I value of 0.7 µg/g. Cd levels, though without universally accepted thresholds, reached up to 0.26 µg/g in Bosa, comparable to values observed in other Latin American urban cohorts. Mn and Cu concentrations were within expected non-occupational ranges. However, it is important to note that reference thresholds may not fully capture population-specific susceptibilities influenced by factors such as age, sex, nutritional status, and metabolic variability (Grandjean et al., 2010; Lucchini et al., 2012).

Spatial contrasts in Pb and Mn concentrations most pronounced in Bosa and Ciudad Bolívar reflect patterns previously reported in Latin American megacities where traffic emissions, mixed land use, and limited green space co-occur (Franco-Rivera et al., 2022; Rodríguez et al., 2015). The moderate Pb/Mn correlation (r = 0.49) suggests partially overlapping sources such as brake wear, tire dust, and resuspended particulate matter (Laidlaw et al., 2012), especially in traffic-dense corridors. These disparities are consistent with environmental justice concerns, as higher metal burdens tend to cluster in socioeconomically disadvantaged areas.

Hg exhibited low and relatively uniform concentrations with only modest elevation in Usaquén. This pattern is consistent with literature indicating that urban Hg exposure is often influenced by diffuse sources particularly diet rather than strictly local emissions (Clarkson & Magos, 2006; Grandjean et al., 1997; 2010). Regional studies similarly report hair-Hg variability linked to broader food systems (Álvarez et al., 2012; Mergler et al., 2007), supporting the interpretation of low, background exposure levels in this context.

Cd and Cu, though less frequently emphasized in Bogotá’s environmental literature, also showed spatial variability. Chronic low-level Cd exposure has been linked to neurocognitive and endocrine effects (Al-Saleh et al., 2013), while Cu imbalance may exacerbate oxidative stress, particularly when co-exposed with other neurotoxicants (Gaetke & Chow, 2003; Zhang et al., 2019). Recent toxicological studies reinforce that such interactions may potentiate redox imbalance and neurodevelopmental effects, especially in early life stages (Khalid et al., 2021; Wang et al., 2022).

The multimetal differentiation captured by PERMANOVA underscores the importance of considering cumulative exposures. Mixture effects may be additive, synergistic, or antagonistic, and single-metal summaries may fail to capture relevant exposure dynamics (Suk et al., 2016).

From a public health standpoint, school-based hair biomonitoring emerges as a cost-effective sentinel tool to detect spatial exposure gradients in complex urban environments. The findings align with the “triple jeopardy” concept, wherein vulnerable populations face overlapping risks from pollution, infrastructural deficits, and limited access to health and social services (Brender et al., 2011). Practical implications include the potential to inform targeted environmental sampling (air, dust, soil), traffic control strategies near schools, and focused biomonitoring in identified clusters before neurodevelopmental harm becomes entrenched. Urban interventions in other megacities such as dust mitigation programs near schools in Mexico City or traffic zoning reforms in São Paulo have shown promise in reducing metal exposure burdens in children (Franco-Rivera et al., 2022; Peña-Fernández et al., 2014).

Several considerations guide interpretation. First, pooled hair sampling enables spatial screening but does not reflect individual variability. Second, hair-metal metrics represent recent cumulative exposure and are sensitive to decontamination and analytical protocols. This study used standardized washing and QA/QC procedures, though the possibility of residual external contamination cannot be excluded. Third, spatial inference is limited to the sampled frame; expanding the sampling network and incorporating temporal dimensions would enhance generalizability. Future work should also integrate environmental matrices (e.g., road dust, soils, particulates) and fine-scale sociodemographic data to isolate emission sources and monitor intervention impacts over time.

### Limitations

The use of pooled hair samples enabled efficient assessment of locality-level exposure patterns but limits inference on within-classroom variability or individual-level distributions. Equal mass contributions per child were used to reduce intra-pool bias; however, demographic heterogeneity (e.g., age or sex structure) may have introduced unmeasured variability. These effects were minimized through standardized random sampling and pooling protocols, though residual bias cannot be entirely excluded.

While rigorous protocols were applied including standardized washing, acid digestion, and QA/QC procedures (blanks, CRMs, calibration, matrix spikes) exogenous contamination and matrix effects remain possible. Analytical uncertainty was greater near element-specific LODs/LOQs, and a small number of triplicates exceeded the ≤ 20% RSD target. These were re-analyzed where possible or reported as qualified. Most matrix spike recoveries were within acceptable ranges (80–120%); however, as detailed in Supplementary Table S8 (formerly S4C), recoveries for Hg fell below this range in two of three replicates (70–92%), indicating potential under-recovery. While cold-vapor AAS with certified hair reference material showed acceptable accuracy, some Hg batches required cautionary flags. Consequently, Hg findings should be interpreted conservatively.

In two Pb batches, reagent blanks slightly exceeded the LOQ; affected samples were re-run or blank-corrected and flagged accordingly. No individual covariates (e.g., diet, seafood intake, hair product use, or recent treatments) were collected, which limits source attribution—especially for Hg—and may introduce residual confounding.

Spatial coverage was restricted to 14 public schools selected purposively to represent a range of urban environmental conditions. Therefore, findings reflect conditions in those localities rather than the entire city. Hair integrates exposure over recent weeks to months but does not capture short-term variation or seasonality. Finally, this was an environmental screening study and was not designed or powered to evaluate health outcomes. Nonetheless, the analytical figures of merit, QA/QC, and replicate precision support the validity of the reported spatial patterns.

## Conclusions

This study applied a spatially resolved hair biomonitoring framework to assess locality-level patterns of Pb, Mn, Cd, Hg, and Cu exposure among schoolchildren in Bogotá. Composite hair samples from 14 public schools across contrasting administrative localities revealed spatial heterogeneity in metal burdens consistent with differential environmental pressures.

Notably, Bosa and Ciudad Bolívar exhibited elevated Pb and Mn, Ciudad Bolívar showed Cd enrichment, and Usaquén presented moderately higher Mn and Hg. Multivariate profiles reinforced these contrasts, identifying clusters for targeted follow-up. The analysis underscores the value of mixture-oriented assessment over single-metal metrics alone in complex urban contexts.

School-based hair biomonitoring emerges as a practical, cost-effective screening tool to detect environmental metal gradients in rapidly urbanizing settings. The results can inform focused public-health actions, including emission control around schools, localized dust mitigation, and enhanced oversight of small-scale industrial activities. Analogous interventions in other megacities such as school dust abatement programs or traffic zoning policies may offer replicable strategies for Bogotá.

Although Hg concentrations were generally low and spatially uniform, matrix-spike recoveries indicated suboptimal analytical performance in two of three replicates. This limitation, discussed in Supplementary Table S8, suggests that true Hg exposure may be slightly underestimated. While the results are consistent with low-level, diffuse urban Hg exposure documented in similar contexts, further validation using higher-sensitivity techniques (e.g., ICP-MS) is recommended for future studies.

Future work should expand the spatial and temporal sampling frame, incorporate environmental matrices (soil, dust, air), and include fine-scale sociodemographic data to refine source attribution, track intervention impact, and assess potential health implications over time.

## Supplementary Information

Below is the link to the electronic supplementary material.

Supplementary file1 (DOCX 369 KB)

## Data Availability

All analytical QA/QC documentation is provided in the Supplementary Information: calibration ranges and regression metrics (Tables S4 and S2), calibration plots and residuals (Figure S1A–B), analyte flow summary chart for metal biomonitoring (Figure S1C), method sensitivity and verification (Table S3), sensitivity analysis for Pb (Table S9), procedural blanks and LOQ checks (Table S5), the batch-level QA acceptance summary (Table S8), certified reference material accuracy (Table S6), and matrix-spike recoveries (Table S7). A completed STROBE checklist is also included (Table S1). De-identified locality-level data tables and the shapefiles used to build the maps are available from the corresponding author upon reasonable request.
