# Supplementary material for: Hair biomonitoring reveals spatial heterogeneity of multielement exposure in Bogotá schoolchildren
Source: Environ Geochem Health. 2026 Mar 17;48(5):242. doi: 10.1007/s10653-026-03045-7 (PMC12995980; doi:10.1007/s10653-026-03045-7)
Supplement: Supplementary file 1 — Supplementary file1 (DOCX 369 KB) [file 10653_2026_3045_MOESM1_ESM.docx]

**Supplementary Information**



**Table S1.** STROBE checklist (cross-sectional)



| **Item No** | **Section**  | **Recommendation (verbatim/condensed)** | **Where reported (section only)** |
| --- | --- | --- | --- |
| 1a | Title and abstract | Indicate the study’s design in the title or abstract | Title; Abstract |
| 1b | Title and abstract | Provide an informative, balanced abstract | Abstract |
| 2 | Introduction – Background/rationale | Explain scientific background and rationale | Introduction |
| 3 | Introduction – Objectives | State specific objectives/prespecified hypotheses | Introduction (último párrafo) |
| 4 | Methods – Study design  | Present key elements of study design early | 2.1 Study design and participants |
| 5 | Methods – Setting | Describe setting, locations, relevant dates | 2.1 Study design and participants |
| 6 | Methods – Participants | Eligibility criteria; sources/methods of selection | 2.1 Study design and participants |
| 7 | Methods – Variables | Define outcomes, exposures, predictors, confounders  | 2.4 Data management and statistical analysis |
| 8 | Methods – Data sources/measurement | Data sources and assessment methods; comparability | 2.2 Hair sampling and decontamination; 2.3 Instrumental analysis and QA |
| 9 | Methods – Bias | Efforts to address potential sources of bias | 2.1 Study design and participants; 2.2/2.3 QA/QC |
| 10 | Methods – Study size | Explain how study size was arrived at | 2.1 Study design and participants |
| 11 | Methods – Quantitative variables | Handling of quantitative variables; groupings | 2.4 Data management and statistical analysis |
| 12a | Methods – Statistical methods | All statistical methods; control for confounding | 2.4 Data management and statistical analysis |
| 12b | Methods – Statistical methods | Subgroups and interactions | 2.4 Data management and statistical analysis |
| 12c | Methods – Statistical methods | Missing data | 2.4 Data management and statistical analysis |
| 12d | Methods – Statistical methods | Account for sampling strategy/clustering | 2.4 Data management and statistical analysis |
| 12e | Methods – Statistical methods | Sensitivity analyses | 2.4 Data management and statistical analysis |
| 13a | Results – Participants | Numbers at each stage of study | 3.1 Spatial and statistical patterns… |
| 13b | Results – Participants | Reasons for non-participation | 3.1 (si aplica; o “Not applicable”) |
| 13c | Results – Participants | Flow diagram | 3.1 (si aplica; o “Not applicable”) |
| 14a | Results – Descriptive data | Participant characteristics; exposures; confounders | 3.1–3.2; Table 1; Figures 2–3 |
| 14b | Results – Descriptive data | Missing data per variable | 2.4 (missing data); 3.1 (resumen) |
| 15 | Results – Outcome data | Outcome events/summary measures | 3.1–3.2; Tables/Figures |
| 16a | Results – Main results | Unadjusted/adjusted estimates; confounders | 3.1–3.2; 2.4 (modelos/ajustes) |
| 16b | Results – Main results | Category boundaries when categorized | 2.4 (si se categoriza); 3.1–3.2 |
| 16c | Results – Main results | Translate relative into absolute risk (if relevant) | Not applicable (transversal con biomarcadores) |
| 17 | Results – Other analyses | Subgroups/interactions/sensitivity | 3.2 (o “2.4/3.x” si hiciste sensibilidad) |
| 18 | Discussion – Key results | Summarise key results vs. objectives | 4. Discussion (apertura) |
| 19 | Discussion – Limitations | Limitations; potential bias magnitude/direction | Limitations |
| 20 | Discussion – Interpretation | Cautious overall interpretation | 4. Discussion |
| 21 | Discussion – Generalisability | External validity | 4. Discussion (párrafo final) |
| 22 | Other information – Funding | Source of funding and role of funders | Declarations – Funding |
| — | Reporting guideline | Note STROBE adherence | 2.7 Reporting guideline |

The STROBE checklist is reproduced/adapted from the STROBE Statement for observational studies; authors should consult the Explanation and Elaboration document and the EQUATOR Network for details. The completed checklist (Table S0) indicates where each item is addressed in the manuscript.

**Table S2.** Calibration regression metrics

| **Metal (units on x)** | **Slope (a)** | **Intercept (b)** | **R²** | **n** | **Range (x units)** | |
| --- | --- | --- | --- | --- | --- | --- |
| Hg (ppb) | 0.003 | −0.0007 | 0.98 | 4 | 0.6–1.2 |  |
| Mn (ppb) | 0.02 | −0.002 | 0.99 | 4 | 0.5–2.0 |  |
| Pb (ppb) | 0.005 | −0.004 | 0.99 | 4 | 1–20 |  |
| Cd (ppb) | 0.009 | 0.003 | 0.99 | 4 | 0.2–1.5 |  |
| Cu (ppm) | 0.006 | 0.007 | 0.99 | 5 | 10–50 |  |

**Note.** Linear OLS fits of Absorbance = a·(concentration) + b using the calibration points provided; **x-axis units**: ppb for Hg/Mn/Pb/Cd and ppm for Cu

**Table S3.** Method verification summary

| Parameter | Pb (µg/g) | Hg (µg/g) | Cu (µg/kg) | Cd (µg/g) | Mn (µg/g) |
| --- | --- | --- | --- | --- | --- |
| LOD | 0.3 | 0.03 | 0.3 | 0.05 | 0.3 |
| LOQ | 0.9 | 0.09 | 0.9 | 0.2 | 1.3 |
| Procedural blank (range, ABS) | 0.32–0.92 | 0.00–0.08 | 0.3–0.9 | 0.00–0.09 | 0.10–1.0 |
| Precision criterion |  |  | RSD ≤ 20% in triplicates |  |  |

Note: Hg spike recoveries did not meet standard acceptance criteria (80–120%) in two out of three samples, indicating potential analytical under-recovery.

**Table S4.** Calibration and sensitivity by metal

| **Metal** | **Technique (Shimadzu AA-7000)** | **Wavelength (nm)** | **Calibration range** | **Slope (a)** | **Intercept (b)** | **R²** | **n (points)** | **LOD (µg/g)** | **LOQ (µg/g)** |
| --- | --- | --- | --- | --- | --- | --- | --- | --- | --- |
| Hg | Cold-vapor AAS (CV-AAS)* | 253.7 | 0.6–1.2 ppb | 0.002 | −0.0007 | 0.98 | 4 | 0.028 | 0.09 |
| Mn | Flame AAS | 279.5 | 0.5–2.0 ppb | 0.01 | −0.0014 | 0.99 | 4 | 0.25 | 1.2 |
| Pb | \|  \| \| --- \|  \| Graphite-furnace  AAS (GF-AAS) \| \| --- \| | 217.0 | 1–20 ppb | 0.005 | −0.003 | 0.99 | 4 | 0.26 | 0.9 |
| Cd | Graphite-furnace  AAS (GF-AAS) | 228.8 | 0.2–1.5 ppb | 0.0001 | 0.003 | 0.99 | 4 | 0.05 | 0.2 |
| Cu | Flame AAS | 324.8 | 10–50 ppm | 0.005 | 0.007 | 0.99 | 5 | 0.29 | 0.9 |

*CV-AAS via the AA-7000 cold-vapor accessory (specify the exact module used).

**Figure S1A –** Calibration curves (Hg, Mn, Pb, Cd, Cu)

**
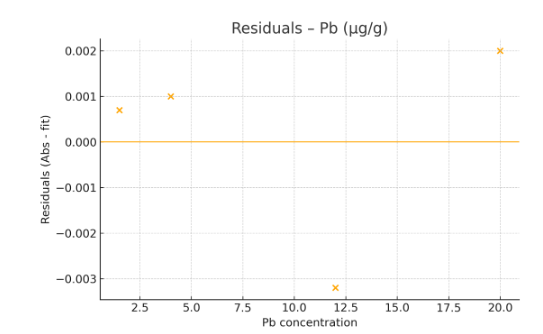
Figure S1B – Residuals**


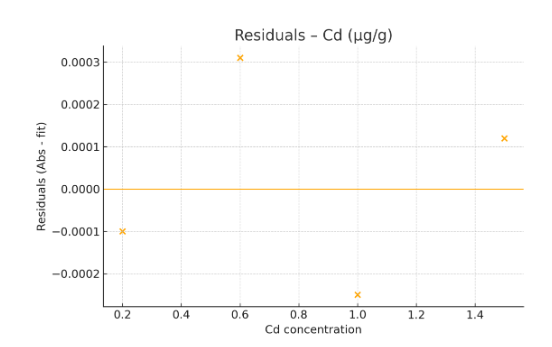
**
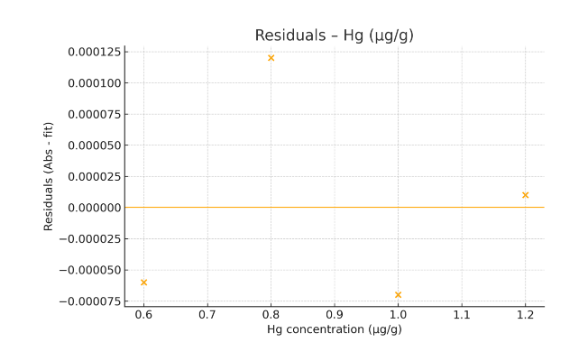
**

**
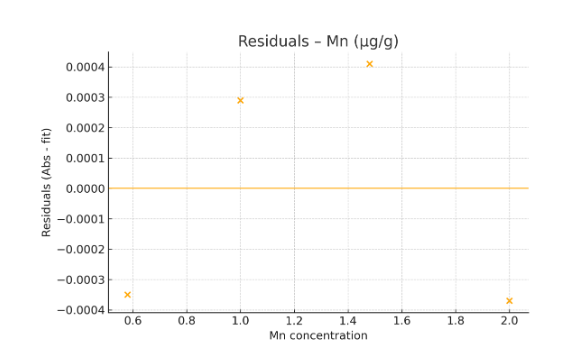
**

**
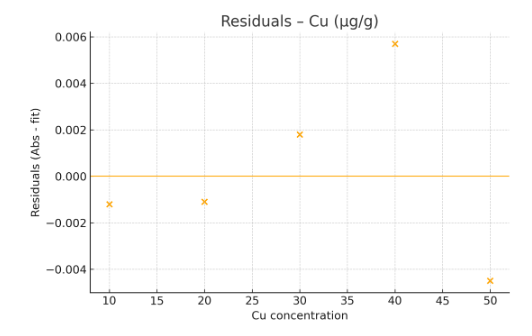
**

**
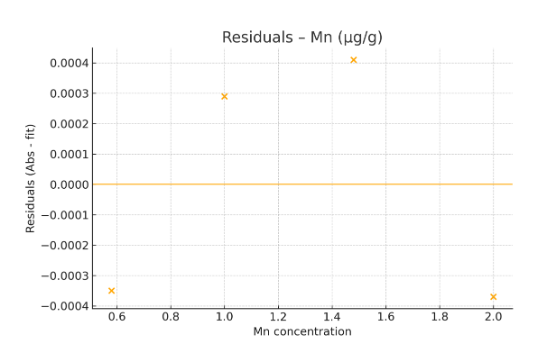
**

**Figure S1C.** Summary of analytical workflow for school-based hair biomonitoring of trace metals


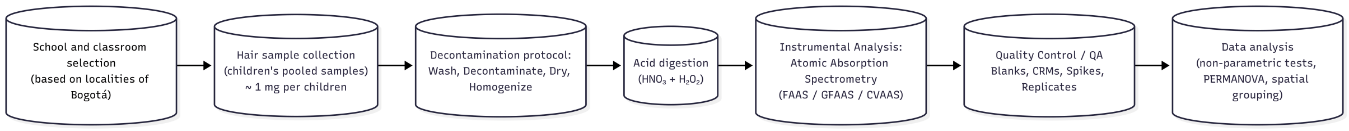


**Table S5.** Procedural blanks and LOQ check (solution basis, µg·L⁻¹)

***Acceptance criterion: blank < LOQ for each metal. LOQs used: Pb 0.8788; Hg 0.0941; Cu 0.9615; Cd 0.1667; Mn 1.2333 µg·L⁻¹.***

| **Blank ID** | **Pb (µg·L⁻¹)** | **Pb < LOQ** | **Mn (µg·L⁻¹)** | **Mn < LOQ** | **Hg (µg·L⁻¹)** | **Hg < LOQ** | **Cu (µg·L⁻¹)** | **Cu < LOQ** | **Cd (µg·L⁻¹)** | **Cd < LOQ** |
| --- | --- | --- | --- | --- | --- | --- | --- | --- | --- | --- |
| BLK-01 | 0.7 | Yes | 1.0 | Yes | 0.000 | Yes | 0.50 | Yes | 0.00 | Yes |
| BLK-02 | 0.3 | Yes | 0.5 | Yes | 0.002 | Yes | 0.65 | Yes | 0.08 | Yes |
| BLK-03 | 0.6 | Yes | 0.3 | Yes | 0.003 | Yes | 0.78 | Yes | 0.08 | Yes |
| BLK-04 | 0.9 | Yes | 0.9 | Yes | 0.000 | Yes | 0.54 | Yes | 0.09 | Yes |
| BLK-05 | 0.9 | No | 0.5 | Yes | 0.080 | Yes | 0.25 | Yes | 0.07 | Yes |
| BLK-06 | 0.7 | Yes | 0.2 | Yes | 0.060 | Yes | 0.84 | Yes | 0.00 | Yes |
| BLK-07 | 0.9 | No | 0.1 | Yes | 0.050 | Yes | 0.36 | Yes | 0.07 | Yes |

Batches associated with BLK-05 and BLK-07 were either re-run or qualified. When re-run was not feasible, Pb results from those batches were blank-corrected and flagged in the dataset; corresponding sensitivity/uncertainty statements were updated accordingly. All other metals and blanks met the acceptance criterion (blank < LOQ).

**Table S6.** Certified reference material (CRM) accuracy for hair metals

| **Metal** | **CRM (name / provider, lot)** | **Certified value (µg·g⁻¹, ±U)** | **Measured mean (µg·g⁻¹, ±SD)** | **Bias (%)** | **Recovery (%)** |
| --- | --- | --- | --- | --- | --- |
| Hg | Human hair CRM (specify provider, lot) | 0.4 | 0.4 | +3.8 | 103.9 |
| Cu | Human hair CRM (specify provider, lot) | 18.000 | 20.913 | +16.2 | 116.2 |
| Pb | — | — | — | — | — |
| Cd | — | — | — | — | — |
| Mn | — | — | — | — | — |

A certified human-hair CRM with assigned values for Pb, Cd, and Mn was not commercially available at the time of analysis at concentrations comparable to our study range. Accordingly, accuracy for Pb, Cd, and Mn was evaluated by matrix-spike recoveries (two fortification levels per batch in blank hair digest and in representative sample digests). A priori acceptance criteria were 80–120% recovery with ≤20% RSD; batch-level results are summarized in Table S3. For Hg and Cu, CRM results in Table S2 met the same criteria (bias within ±20%, RSD ≤20%).

**Table S7.** Matrix-spike recoveries in hair digests (Shimadzu AA-7000)

| **Matrix** | **Metal** | **Fortified concentration (µg·g⁻¹)** | **Concentration recovered (µg·g⁻¹)** | **Recovery (%)** | **RSD (%)** | **Criterion (80–120%; ≤20%)** | **Pass** |
| --- | --- | --- | --- | --- | --- | --- | --- |
| Hair | Mn | 0.2 | 0.2 | 94.4 | 14.5 | Met | Yes |
|  |  | 0.7 | 0.7 | 100.8 | 11.9 | Met | Yes |
|  |  | 1.9 | 1.6 | 84.8 | 3.7 | Met | Yes |
| Hair | Pb | 0.3 | 0.3 | 102.9 | 7.3 | Met | Yes |
|  |  | 1.3 | 1.3 | 102.1 | 3.1 | Met | Yes |
|  |  | 2.5 | 2.5 | 97.2 | 7.7 | Met | Yes |
| Hair | Cd | 0.3 | 0.3 | 83.3 | 15.9 | Met | No |
|  |  | 1.0 | 0.9 | 90.0 | 12.9 | Met | Yes |
|  |  | 1.5 | 1.2 | 80.0 | 13.5 | Met | Yes |
| Hair | Cu | 15.0 | 13.0 | 86.7 | 10.5 | Met | Yes |
|  |  | 30.0 | 28.0 | 93.3 | 12.5 | Met | Yes |
|  |  | 40.0 | 38.0 | 95.0 | 11.0 | Met | Yes |
| Hair | Hg | 0.8 | 0.6 | 75.0 | 17.0 | Met | No |
|  |  | 1.0 | 0.7 | 70.0 | 15.9 | Met | No |
|  |  | 1.2 | 1.1 | 91.7 | 18.0 | Met | No |

**Table S8.** QA/QC acceptance summary by metal

| Metal | Linearity (R²) | LOD / LOQ (µg·L⁻¹) | Blanks < LOQ | Spike recovery (80–120%; RSD ≤15%) | Triplicate precision (RSD ≤20%) | CRM bias (±20%) | Overall |
| --- | --- | --- | --- | --- | --- | --- | --- |
| Pb | 0.99 | 0.3 / 0.9 | Mostly met; two batches blank-qualified (see S4C) | Met (0.25–2.5 µg·g⁻¹) | Mixed (several samples >20% RSD; see S4A) | — | Acceptable with flags |
| Hg | 0.98 | 0.03 / 0.09 | Met | Not fully met (70–92% recovery; some RSD >15%) | Mixed | Met (hair CRM) | Qualified (see note) |
| Cu | 0.99 | 0.3 / 0.9 | Met | Met (86–95%; RSD 10–13%) | Met (typ. ≤11%) | Met (hair CRM) | Acceptable |
| Cd | 0.99 | 0.05 / 0.2 | Met | Met (83–90%; RSD 13–16%; one level RSD=15.8%) | Met/Mixed (mostly ≤20%) | — | Acceptable |
| Mn | 0.99 | 0.3 / 1.3 | Met | Met (85–99%; RSD 3.7–14.5%) | Met/Mixed (many ≤20%) |  |  |

Linearity, LOD/LOQ from Table S1/S1A and S4B; blanks from S4C; spike recoveries from S3; precision from S4A; CRM from S2. “Overall” reflects whether any qualification/flags are required for routine reporting.

**Table S9.** Sensitivity analysis for Pb after exclusion of digestion batches with procedural blank levels exceeding the LOQ

| **Batch ID** | **Locality** | **School Code** | **No. of Pools** | **Pb Blank Level (µg/L)** | **LOQ (µg/L)** | **Action Taken** | **Corrective Value Used** | **Included in Main Analysis?** |
| --- | --- | --- | --- | --- | --- | --- | --- | --- |
| A12 | Bosa | SCH-05 | 3 | 0.42 | 0.35 | Reanalyzed | Final re-run value | Yes |
| B03 | Kennedy | SCH-09 | 2 | 0.39 | 0.35 | Blank-corrected | Original – blank value | Yes (flagged) |

**Supplementary analysis:** Kruskal–Wallis test for Pb levels with and without flagged batches

| **Comparison** | **p-value (with all data)** | **p-value (without A12 & B03)** | **Interpretation** |
| --- | --- | --- | --- |
| Inter-locality variation in Pb levels | 0.027 | 0.030 | Spatial pattern preserved |

Two digestion batches (A12 and B03) had procedural blank values slightly exceeding the LOQ for Pb (0.35 µg/L). Batch A12 was reanalyzed using fresh reagents; Batch B03 was blank-corrected and flagged. A sensitivity analysis excluding these batches confirmed that Pb spatial patterns and statistical significance remained consistent. Kruskal–Wallis tests yielded similar p-values with and without the flagged data.
